# Alignment in Multimodal Interaction: An Integrative Framework

**DOI:** 10.1111/cogs.12911

**Published:** 2020-10-29

**Authors:** Marlou Rasenberg, Asli Özyürek, Mark Dingemanse

**Affiliations:** ^1^ Centre for Language Studies Radboud University; ^2^ Max Planck Institute for Psycholinguistics; ^3^ Donders Institute for Brain, Cognition and Behaviour Radboud University; ^4^ Communicative Alignment in Brain and Behaviour Team Language in Interaction Consortium

**Keywords:** Social interaction, Alignment, Mimicry, Behavior matching, Accommodation, Entrainment, Co‐speech gestures

## Abstract

When people are engaged in social interaction, they can repeat aspects of each other’s communicative behavior, such as words or gestures. This kind of behavioral *alignment* has been studied across a wide range of disciplines and has been accounted for by diverging theories. In this paper, we review various operationalizations of lexical and gestural alignment. We reveal that scholars have fundamentally different takes on when and how behavior is considered to be aligned, which makes it difficult to compare findings and draw uniform conclusions. Furthermore, we show that scholars tend to focus on one particular dimension of alignment (traditionally, whether two instances of behavior overlap in form), while other dimensions remain understudied. This hampers theory testing and building, which requires a well‐defined account of the factors that are central to or might enhance alignment. To capture the complex nature of alignment, we identify five key dimensions to formalize the relationship between any pair of behavior: time, sequence, meaning, form, and modality. We show how assumptions regarding the underlying mechanism of alignment (placed along the continuum of *priming* vs. *grounding*) pattern together with operationalizations in terms of the five dimensions. This integrative framework can help researchers in the field of alignment and related phenomena (including behavior matching, mimicry, entrainment, and accommodation) to formulate their hypotheses and operationalizations in a more transparent and systematic manner. The framework also enables us to discover unexplored research avenues and derive new hypotheses regarding alignment.

## 1 Introduction

In social interactions, people coordinate their actions in an effort to incrementally and interactively reach their communicative goals. One component of such joint actions is cross‐participant repetition of communicative behavior. Work across a wide range of fields shows that when people are engaged in communicative interaction, their behaviors may grow to be in tune with each other at several levels: from body postures and eye gaze, to words and gestures. A key research objective within cognitive science is to gain a fuller understanding of this kind of behavioral *alignment* and how this can lead to mutual understanding. To answer this question, we benefit from adopting a broad perspective, by also considering work on related concepts, such as behavior matching, imitation, mimicry, entrainment, repetition, and accommodation (which may serve other, partially overlapping cognitive or socio‐affective functions).

To get a grip on the phenomenon of alignment, we need to start from the vantage point that natural communication is inherently multimodal, comprising both speech and such bodily behaviors as facial expressions, eye gaze, and co‐speech gestures. *Co‐speech gestures* are meaningful movements (usually of the hands or arms) that accompany speech. A subset of these are so‐called *iconic* gestures, which visually depict object attributes, spatial relationships, or actions. Consider the following example from *The Late Late Show* with James Corden (an American late‐night talk show). The talk show guests, Mila Kunis (M) and Christian Slater (C), are engaged in a conversation about the dating show *T* *he Bachelorette*. In this show, one particular participant (“Chad”) became known for always eating meat on camera.

**Table:** 

(1)		C:	Do you remember how crazy Chad was in that one sea‐
		M:	The **meat [eating]_M_ Chad**?
		C:	Yeah the **meat [eating]_C_ Chad** guy

**Fig. 1:**
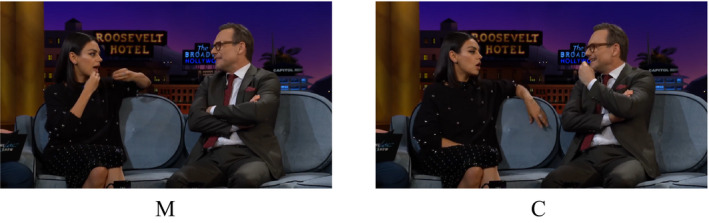
Alignment of speech and gestures produced by the talk show guests Mila Kunis (M) and Christian Slater (C).^1^

Square brackets indicate the start and end points of a gesture, and the capital letters correspond to the pictures shown in Fig. 1. In this excerpt, M uses the lexical phrase “meat‐eating Chad” along with an iconic co‐speech gesture depicting the act of eating. C repeats both the lexical phrase (“meat‐eating Chad”), as well as the eating gesture.^2^ This kind of lexical and gestural alignment occurs regularly in both natural and task‐based interactions, and it has been shown to support joint problem‐solving and coordination (e.g., Holler & Wilkin, 2011; Pickering & Garrod, 2004).

Despite the emergence of various theoretical accounts, a comprehensive understanding of the phenomenon of alignment is still lacking. This is partly due to the large variation in methodological approaches. Take again Example 1 above. We can easily identify some form of alignment here in that both participants produce the same lexical phrase (“meat‐eating Chad”) and similar‐looking gestures. This focus on alignment of form ties in with the traditional notion of behavioral alignment. However, in order to have a complete understanding of the phenomenon—when, how, and why it happens—there are other dimensions to consider. For example, some scholars quantify the extent to which the spoken utterances or gestures overlap in form, while others care more about the fact that both speakers used similar words or gestures to collaboratively refer to the same person. Some restrict analyses to alignment in speech *or* gestures, while others look at both. Some only focus on cases of alignment in adjacent speech turns, while others also look for alignment of behaviors which are further apart in time. Design choices and measurement techniques vary both across and within fields, and they often (implicitly) follow from theoretical presuppositions. This makes it difficult to bring the findings together into an all‐encompassing view of why and how alignment comes about for various types of behavior in interactions.

Given the diversity of work in the interdisciplinary area of social interaction, some notes on terminology are in order. First, scholars have used the term “alignment” in different ways. In the most general sense, in the context of social interaction, alignment could be taken to mean interpersonal coordination between two communicators. The term (interactive) alignment was originally introduced by Pickering and Garrod (2004) to refer to the interpersonal alignment of mental representations underlying linguistic behavior. However, various scholars have used the same term to simply refer to observable similarities in communicative behavior itself (e.g., Bergmann & Kopp, 2012; Fusaroli et al., 2017; Howes, Healey, & Purver, 2010; Oben & Brône, 2016). Of course, the two senses are related (since inferences about mental representations are often made on the basis of observed behavior), but in light of theoretical discussions, it is important to keep them apart. Therefore, we differentiate between *behavioral alignment* and *alignment of mental representations*. Most of the empirical work reviewed in this paper is concerned with behavioral alignment. So we use terms like lexical alignment and gestural alignment with the intuitive meaning that two people produce similar lexical items or phrases or co‐speech gestures (similar to the discussion of the example above). We make it explicit when we are referring to alignment of mental representations instead.

Second, what we call behavioral alignment here has been studied under a range of terms, and it is part of a larger array of phenomena variously labeled behavior matching, entrainment, accommodation, repetition, imitation, and mimicry. Though all of these terms target contingent behavioral similarities in socially interacting agents, each of them comes with its own disciplinary history. Thus, each carries its own commitments and implications with regard to the kinds of behavior in focus, the embodied and interactional mechanisms at play, and the cognitive or socio‐affective functions involved. While we opt for “alignment” as a widely used and relatively theory‐agnostic term, a key contribution of our paper is to provide an integrative framework that can enable cumulative progress regardless of the precise label used.

With many fields now working toward empirical and theoretical accounts of alignment, it is crucial to have a shared framework that allows us to capture the space of possibilities of what can be considered alignment. By systematically tracking five dimensions along which communicative behaviors may relate to each other, we formulate clear and unambiguous terms of comparison that help to sharpen and contrast predictions of different theoretical approaches. We illustrate the utility of this framework by reviewing recent and foundational work on lexical and gestural alignment. Our approach makes visible how methodological choices and operationalizations tend to pattern together with assumptions regarding underlying mechanisms (for instance, *priming* vs. *grounding*), resulting in a situation where some areas of the space of possibilities are much better explored than others. We devote special attention to the interrelation of lexical and gestural alignment as one of the promising areas for future studies.

## 2 Theoretical approaches to alignment

Social interaction is an incredibly complex process, which has resulted in a diverse set of empirical and theoretical approaches. In the field of alignment, however, theoretical contributions are usually framed as belonging to one of two prominent “camps,” which could be denoted as *priming* and *grounding* (cf. Oben, 2018; also denoted automatic vs. strategic alignment [Kopp & Bergmann, 2013]; and related to the distinction between Aggregate and Interactive approaches [Healey, Mills, Eshghi, & Howes, 2018]). According to this dichotomy, priming accounts suggest that alignment involves an automatic, low‐level priming mechanism that is confined to the individual’s mind (e.g., Pickering & Garrod, 2004, 2006), whereas grounding accounts argue that alignment follows from interactive, coordinative efforts involved in joint meaning‐making (Brennan & Clark, 1996; Holler & Wilkin, 2011).

The priming versus grounding juxtaposition falls short in two respects. First, because the accounts are not mutually exclusive, and second, because it does not do justice to the wealth of integrative theory on communication more generally (also beyond the specific phenomenon of alignment). Nonetheless, as shall be seen, empirical investigations of alignment often appear to be implicitly guided by either of the two perspectives. We will discuss these perspectives against the backdrop of a more inclusive set of theories on social interaction from various fields relevant to the study of alignment. We find that theories differ from one another on two key aspects: (a) the extent to which they presume perspective‐taking, and (b) the relation they predict between alignment at various levels.

In some theories, cross‐participant repetition of communicative behavior is not considered to be produced “for’ the conversational partner or with the partner’s perspective in mind. For example, according to *direct mapping accounts*, the partner’s behavior directly activates the corresponding motor representations (through the mirror neuron system), which underlies the production of the same behavior (e.g., Brass & Heyes, 2005; Dijksterhuis & Bargh, 2001; Heyes, 2011; Rizzolatti, Fogassi, & Gallese, 2001).^3^ In a similar vein, the *interactive alignment account* (Pickering & Garrod, 2004) entails a parity between the representations used in comprehension and production, and therefore hearing a certain phoneme, word, or syntactic structure (which leads to the activation of the corresponding representation) “primes” the hearer to subsequently use it in his/her own speech production as well. “As part of this process, interlocutors do not model each other’s mental states but simply align on each other’s linguistic representations” (Pickering & Garrod, 2004, p. 180).

On the other end of the continuum are theories in which communicators carefully keep track of and adjust to their partner’s perspective. For example, Clark et al. argue that people explicitly represent the information that is shared (and mutually known to be shared) with the communicative partner; that is, they keep track of their *common ground* (Clark, 1996). Using an object description task, it has been shown that people establish partner‐specific shared conceptualizations of objects that become part of common ground (e.g., conceptualizing a particular shoe as a “loafer”; Brennan & Clark, 1996). Communicators repeatedly refer to these *conceptual pacts* with the same words when talking to the same partner, thus yielding sustained lexical alignment (or using the original term: “lexical entrainment”), which they abandon when switching to another partner with whom the pact is not shared.

We could conceptualize priming versus grounding as being positioned on either end of a continuum, as they represent opposing ideas on the involvement of perspective‐taking in alignment (see also the discussion of “mediated” vs. “unmediated” accounts of alignment in Branigan, Pickering, Pearson, McLean, & Brown, 2011). However, there are also theories that assume more moderate perspectives. Such theories argue that having to always explicitly represent and fully adopt to the partner’s perspective might be too costly in terms of cognitive resources, but it is necessary to some extent or under some special circumstances. One such proposal is the idea that (language) processing takes place in two “stages”: an early egocentric phase, followed by a later phase in which one might correct for the partner’s perspective (e.g., Horton & Keysar, 1996; Keysar, Barr, & Horton, 1998; Lin, Keysar, & Epley, 2010). In contrast, Brennan, Galati, and Kuhlen (2010) propose that communicators do engage in partner‐adapted processing early on. However, they argue that rather than this resulting from a detailed representation of the partner’s perspective, communicators make use of a simplified model, such as “my partner knows X” or “my partner does not know X” (so‐called one‐bit partner models).

Coming to the second key aspect, namely the level(s) at which alignment is presumed to take place, it should first be noted that both priming and grounding approaches are concerned with alignment of behavior (called *repetition* or *entrainment*) as well as alignment of higher level mental representations (*situation models* or *conceptual pacts*). However, they differ in how they theorize alignment at distinct levels. According to priming accounts, speakers do not only observably align their speaking behavior, but also the linguistic representations underlying that behavior: “they have aligned linguistic knowledge to the extent that they have similar patterns of activation of linguistic knowledge” (Pickering & Garrod, 2006, p. 215). Furthermore, the priming mechanism is argued to operate at multiple linguistic levels (from phonetics to semantics), where alignment at one level leads to alignment at other levels, ultimately resulting in alignment of situation models.

In approaches taking a grounding perspective, alignment of linguistic representations is not a requisite for alignment at other levels of representation. Conceptual pacts are formed through a process of *grounding* interactional contributions (Brennan & Clark, 1996; Clark & Brennan, 1991), which can happen in various ways. For example, alignment can be used to signal understanding, thereby grounding a certain referring expression (as could be argued for the “meat‐eating Chad” in Example 1). However, alignment of communicative behavior can also occur in the form of other‐initiated repair, thus signaling *misunderstanding* as a means to *get to* higher level alignment, rather than being an indicator of it (e.g., Mills & Healey, 2008), as in the following example from Clark and Wilkes‐Gibbs (1986):

**Table:** 

(2)	A	Uh, person putting a shoe on.
	B	Putting a shoe on?
	A	Uh huh. Facing left. Looks like he’s sitting down.
	B	Okay.

Furthermore, (purposefully) using different words or gestures can also be a way to establish mutual understanding (e.g., Clark & Wilkes‐Gibbs, 1986; Holler & Wilkin, 2011; Tabensky, 2001). For example, Holler and Wilkin (2011) describe a situation where participant A referred to a figure with the lexical phrase “an ostrich,” to which participant B replied, “Yeah, okay that, that looks like a woman to me, kicking her leg up behind her, yeah?’’ (though interestingly both produced the same gesture along with the speech, as further discussed in Section 4.5). Using the terminology of Clark and Wilkes‐Gibbs (1986): The presentation of participant A was not accepted by participant B, who used the repair strategy *replacement* in an effort to get to a shared conceptualization of the figure.

Thus, in grounding accounts there is a flexible relationship between behavioral alignment (in various modalities or linguistic levels) and alignment of conceptual representations, while for priming accounts this is presented as causally linked, with alignment percolating across all levels.

In general, in the cognitive sciences, cross‐participant repetition of communicative behavior has been theorized as involving shared representations—be they shared linguistic or conceptual representations as just discussed, or shared motor (Rizzolatti et al., 2001), goal (Bekkering, Wohlschläger, & Gattis, 2000; Wohlschläger, Gattis, & Bekkering, 2003), or task representations (Sebanz, Bekkering, & Knoblich, 2006). Yet there is a class of theories that attempts to account for human interaction without appealing to mental representations, namely dynamical systems theory (for an insightful overview, see Dale, Fusaroli, Duran, & Richardson, 2013). For example, Shockley, Richardson, and Dale (2009) propose that interpersonal coordination can be thought of as a “coordinative structure—a self‐organized, softly assembled (i.e., temporary) set of components that behave as a single functional unit” (p. 313), which does not necessarily involve higher level cognitive representations. This means that when talking about alignment, it is important to first of all distinguish empirically observable alignment of behavior from the presumed alignment of mental representations. And for the latter, to differentiate between alignment of various kinds of representations (motor, linguistic, etc.), as theories make different claims about their involvement and interrelations in social interaction.

## 3 A framework for understanding and investigating alignment

In order to go beyond these existing theoretical approaches, we have to outline the space of possibilities of how alignment is conceptualized and measured across studies. Generally speaking, all studies of alignment compare behavior from person A with behavior from person B. These behaviors can be discrete events (e.g., one gesture) or streams of behavior (e.g., a series of consecutive body movements). When these behaviors are aligned, they are considered to be “the same” or “matched” in one way or the other. That is, the units of analysis are cross‐participant paired behaviors, where A’s behavior is similar to B’s behavior on one or several dimensions. The term “prime‐target pairs” is commonly used in controlled experiments on alignment. We use *paired behaviors* here as a more neutral term that does not presuppose a particular methodological or theoretical approach and is agnostic about the mechanism behind the pairing.

Empirical studies show considerable variation with respect to the dimension(s) they take into consideration, and how they operationalize alignment. Most studies use similarity in form as a criterion for alignment, with various definitions and measures of form overlap. However, the relation between the two instances of behavior on other dimensions is often taken for granted or ignored. This is problematic, as this is where theoretical approaches might have diverging hypotheses. In order to move forward in the field, we need a tool to sharpen and contrast predictions of different theoretical approaches, and to operationalize experimental studies accordingly.

**Fig. 2:**
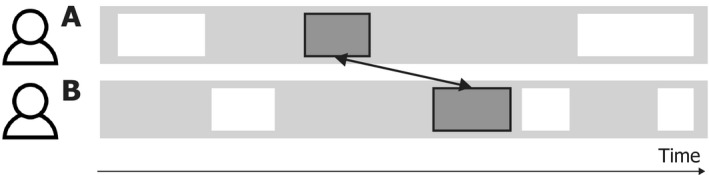
Visualization of an interaction between two people. Every rectangle represents an instance of behavior. The behavior can be of various types (i.e., the rectangles could represent syntactic constructions, lexical choices, mannerisms, co‐speech gestures, etc.) and units of analysis (e.g., the rectangles could represent discrete events or a stream of behavior). The arrow indicates a possible comparison between two instances of behavior.

In an effort to clarify and reveal (oftentimes implicit) differences in what is considered to be aligned, we introduce a common integrative framework to decompose the notion of behavioral alignment into its constituent dimensions. We consider five key dimensions that help characterize the relation between any pair of behaviors: time, sequence, meaning, form, and modality. The framework is presented below, where we outline the dimensions in terms applicable to all kinds and levels of verbal and nonverbal behavioral alignment, be it posture or gesture, phonetics or syntax. For illustrative purposes, we use rectangular shapes as instances of behavior, which are produced by two interlocutors (A and B), as shown in Fig. 2.

**Table 1:** A multidimensional framework for understanding and investigating alignment^a^

Time	The temporal distance between the first and second part of a pair of behavior can be a short interval (e.g., simultaneous production or a split‐second delay) or a long interval (varying from one or multiple turns, several minutes, or even hours)	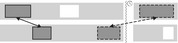
Sequence	The sequential relation between any pair of aligned behavior can vary from occurring within a certain sequence (e.g., the behavior occurs within the same trial, as indicated by the larger rectangles in the figure), to transcending such sequential boundaries	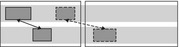
Meaning	For levels of behavior which convey meaning (e.g., lexical items or gestures), any pair of behavior can vary from conveying the same meaning or referent to conveying different meanings	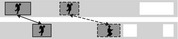
Form	The two parts of a pair of behavior can vary from being exact copies, to having little or no overlap in form or shape	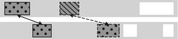
Modality	The two parts of a pair of behavior can be produced in the same modality (e.g., the two pair parts are both spoken sentences), but can also be produced in different modalities (e.g., the first pair part is a lexical phrase, and the second pair part an iconic gesture)	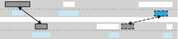

^a^ The relationship between the two parts of a behavior pair can vary on five dimensions, as outlined in this table. For each dimension, we visualize two different relationships between instances of behavior—one with a solid arrow and one with a dashed arrow. For meaning, we use tangram figures to visualize the referent of speech and/or gestures (cf. Clark & Wilkes‐Gibbs, 1986; Holler & Wilkin, 2011).

We consider the five dimensions to be inherent to all kinds of paired behaviors. The relation between any two behaviors (or streams thereof) can always be described and analyzed in terms of time, sequence, meaning, form, and modality. In the following, we outline what it means for paired behaviors to be related on these five dimensions, and we explain how the dimensions can be employed in empirical studies (Table 1).

First, behaviors have a relation in terms of *time*. The temporal lag between paired behaviors can vary from none (in the case of simultaneous production), to a delay of several (milli)seconds or minutes (e.g., as a result of intervening filler trials; Hartsuiker, Bernolet, Schoonbaert, Speybroeck, & Vanderelst, 2008), and can go up to hours or even days. When dealing with multiple streams of behavior, one can also observe the temporal relation of those time series, for example, in terms of synchrony or convergence in multiscale clustering (Abney, Paxton, Dale, & Kello, 2014).

Second, paired behaviors may or may not occur in a conversational *sequence*. A key property of human interaction is that participants take turns, where each turn has a particular sequential relation to a prior turn in the discourse. A clear example of this are “adjacency pairs”; pairs of utterances where the latter is functionally dependent on the first, such as offer–acceptance or question–answer (Schegloff & Sacks, 1973). At a higher level, one or several of such pairs together can constitute a course of action (Levinson, 2013; Schegloff, 2007), such as scheduling a meeting. In a similar vein, task‐based interactions can have an experimentally imposed sequential structure in terms of games (e.g., in the Maze task; Garrod & Anderson, 1987) or trials (e.g., in picture description tasks; Branigan, Pickering, & Cleland, 2000; or referential communication tasks; Holler & Wilkin, 2011). The sequence dimension captures the fact that paired behaviors in a question–answer sequence have a different relation to each other than, say, paired behaviors across experimental trials.

Third, paired behaviors can be related to one another in terms of their *meaning*. Here the type of behavior plays an important role, as it is more meaningful to talk about whether people mean the same thing with a particular word they utter (Garrod & Anderson, 1987), compared to say, the pitch or foot‐wiggles they produce. Furthermore, it is important to note here that we are moving into the domain of alignment of mental representations (as we cannot empirically observe semantics or reference directly), rather than alignment of visible (and directly measurable) characteristics.

Fourth—and this is the most intuitive and well‐studied dimension—paired behaviors can be more or less similar in *form*. For example, one could measure the (dis)similarity in the syntactic composition of two utterances (Reitter & Moore, 2014), the extent to which two spoken words have similar acoustic attributes (Pardo, Urmanche, Wilman, & Wiener, 2017), or whether two body movements are contra‐ or ipsilateral (Bavelas, Black, Chovil, Lemery, & Mullett, 1988).

Finally, paired behaviors can occur in the same or in a different communicative *modality*. For example, lexical items produced in the spoken modality could be compared to other lexical items in the written modality, or to co‐speech gestures (Tabensky, 2001) or facial expressions (Bavelas, Coates, & Johnson, 2000) produced in the visual modality. The dimension of modality captures the mode in which paired behaviors are produced and interpreted.

Though it is clear that studies have operationalized alignment in different ways, our framework makes visible that sometimes they have focused on different dimensions altogether, or have applied them in fundamentally different ways. Any particular dimension can be used as a *grouping criterion* or as a *measurement variable* in an empirical study. For example, one might restrict analyses to adjacent behaviors or adjacent speech turns, and quantify the extent to which they overlap in terms of form (i.e., sequence as grouping criterion, form as a measurement variable; e.g., Bergmann & Kopp, 2012; Fusaroli et al., 2017). Or one could search for all behavior of a particular form and modality, and quantify their temporal relations (i.e., form and modality as grouping criterions, time as a measurement variable; e.g., Louwerse, Dale, Bard, & Jeuniaux, 2012). Clearly, although all of these studies can be described as investigating “alignment,” the operationalizations are so different that one may question their commensurability. One goal of our framework is to make it more straightforward to pinpoint similarities and differences.

Some of the dimensions are interrelated. For example, when two instances of behavior occur within a certain sequence (e.g., in a question–answer pair), this naturally has consequences for the temporal relation (i.e., the two pair parts are likely to have only a short temporal lag). However, it is possible to experimentally tease them apart, for example, by manipulating the presence of intervening material between a question (prime) and an answer (target), thereby increasing the temporal lag while retaining sequential cohesion (Levelt & Kelter, 1982). Another interdependency becomes apparent when comparing instances of behavior which are produced in different modalities (e.g., a lexical phrase is compared to an iconic gesture), as here the dimension of form will become less relevant. Due to these interrelations, certain dimensions can become conflated or taken for granted in both empirical and theoretical approaches. Yet it is crucial for work on alignment to treat the dimensions as conceptually distinct from each other and to specify the relationship between two instances of behavior for each dimension separately. We will corroborate this in the next section, in which we apply the framework to studies on lexical and gestural alignment.

## 4 A review based on the framework

This section will illustrate how we can use the five dimensions introduced in the previous section to characterize and compare previous studies on alignment in a systematic manner. We will start each subsection by reviewing the range of empirical possibilities for incorporating that dimension when studying alignment, and we will conclude each section by discussing how these operationalizations relate to the two theoretical approaches (priming and grounding). By doing so, we will show which dimensions are of fundamental importance in various empirical and theoretical accounts, and which dimensions are understudied. We will zoom in on lexical and gestural alignment, though in essence this practice can be applied to work on alignment at all linguistic levels or kinds of behavior, making the current discussion of relevance to the field as a whole.

We will restrict our focus to studies investigating spontaneous, interactive dialogs (free conversations or task‐based), thus excluding studies with interactions which are (partly) scripted, or lack natural turn‐taking and feedback (e.g., Kimbara, 2008; Mol, Krahmer, Maes, & Swerts, 2012). Moreover, we will narrow the focus to studies on lexical alignment at the word level (thus excluding alignment of syntax or phonology, as well as higher level pragmatic levels, such as dialog acts, e.g., Louwerse et al., 2012) and co‐speech gestures (thus excluding bodily behavior such as posture, e.g., Chartrand & Bargh, 1999). Note that this is not intended to be a complete review of all studies in the field, but instead an illustration of the range of empirical and theoretical approaches for studying alignment, and how they can be positioned in the overall possibility space.

### 4.1 Time

The time dimension can be used as a grouping variable by defining a particular temporal lag between aligned pairs of behavior. In (priming) experiments, such lags can be experimentally controlled, for instance, by varying the amount of fillers items that appear between prime and target (Mahowald, James, Futrell, & Gibson, 2016). In corpus studies, alignment could be operationalized as having to occur within a predefined temporal window. A useful technique for the latter approach is that of *time‐aligned moving averages* (TAMA), where a specific time‐window (e.g., of 40 s) is shifted across the time axis in a stepwise manner. However, this has mostly been used for analyses of prosody (e.g., De Looze, Scherer, Vaughan, & Campbell, 2014), and it is not common for lexical or gestural alignment (but see Oben, 2015).

In contrast, there are studies where for a gesture or lexical pair to count as aligned, there are no restrictions on the amount of time which can intervene. These are typically qualitative studies, which use a descriptive or exploratory approach (e.g., Kimbara, 2006; Tabensky, 2001; Tannen, 1989), though it also applies to some quantitative studies (e.g., Holler & Wilkin, 2011).

The importance of methodological choices regarding time restrictions might be downplayed, because alignment often occurs with a split‐second delay or is intervened by one or a few turns, which means that both approaches will yield a highly similar selection of cases. However, the paired behaviors that are part of the analyses can still differ considerably across studies: Whereas in the work by Oben (2015) only gestures that occurred within a window of 40 s were considered for alignment, in the study by Holler and Wilkin (2011) the gestures could be as far apart as several minutes (though the actual time lags are not reported), as long as they were referring to the same referent (see Section 4.3).

Instead of selecting candidate paired behaviors based on a preset time window, one can also measure the overall temporal coupling of two streams of behaviors and determine temporal lag more dynamically. For example, Louwerse et al. (2012) analyzed the multimodal interactions of participants engaged in a route communication task (Map Task; cf. Anderson et al., 1991). They investigated the temporal dependencies of “matched” verbal, facial, and gestural behaviors using Cross Recurrence Quantification Analysis (for a discussion of this method, see Fusaroli, Konvalinka, & Wallot, 2014). This yielded average time intervals per behavior category, such as 25 s for deictic (i.e., pointing) gestures. Going beyond such analyses of synchronization, it also possible to measure convergence in multiscale clustering of behavioral events. To our knowledge, this has not yet been applied to lexical or gestural behavior, but there is promising work that captures the temporal clustering of speech acoustics using power law distributions (Abney et al., 2014).

How the dimension of time is used often relates in complex ways to one’s theoretical assumptions and hypothesized mechanisms. Alignment across large time intervals is less likely to be considered in studies working from a priming approach, as priming effects are hypothesized to decrease over time.^4^ From a grounding perspective, a similar prediction can be made for natural interactions, given that topics vary over the course of interactions, thereby decreasing the relevance of certain conceptual pacts and the need to keep repeating certain lexical items or gestures. However, with respect to the grounding perspective, interlocutors have also been shown to repeat words after long temporal lags in free conversations, for example to reintroduce a topic or tie back to a problematic turn which was produced earlier in the conversation (Dingemanse, Blythe, & Dirksmeyer, 2014; Sacks, 1992; Schegloff, 2000). Thus, in general, both theoretical accounts would argue that over time, the likelihood of encountering behavioral alignment decreases, though for priming this effect would be mechanistic in nature (due to decreased levels of activation), while for grounding it would be more incidental (related to changes in joint projects and topics).

In addition to considerations regarding (the lack of) restrictions on the *maximum* time interval, the *minimum* time interval is also relevant. Words or gestures are sometimes produced simultaneously by two speakers, for example, when they interrupt each other or co‐produce an utterance (cf. Holler & Wilkin, 2011; Tannen, 1989), which is a well‐documented phenomenon in conversation analysis (e.g., Lerner, 2002). Yet besides a methodological challenge, such cases are also a challenge for theoretical accounts based on priming as the underlying mechanism (if the particular word or gesture had not yet been produced prior to that moment). Such cases might be better explained from the grounding perspective, coupled with an account of incremental and predictive sentence processing.

### 4.2 Sequence

Paired behaviors do not merely stand in a temporal relation to one another; often they also occur within or across larger conversational sequences. The sequence dimension has been used as both a grouping and measurement criterion in studies on lexical and gestural alignment. At the outset, sequence can be employed to define which part of an interaction will be included in the analysis. For example, Chui (2014) qualitatively investigated gestural alignment in 12 short stretches of talk in free interaction, in which people communicated about the meaning of a referent. Thus, analyses were restricted to co‐speech gestures that were produced in a specific conversational sequence. Alternatively, in quantitative studies, conversations have also been studied as one large chunk, without the differentiation into sequences. For example, Bergmann and Kopp (2012) compared all iconic and deictic gestures from 25 dyads engaged in a spatial communication task (alternating direction‐giving and sight description), yielding a total of 3,993 cross‐participant gesture comparisons for the analyses.

Once the to‐be‐analyzed data have been selected, a possible approach is to look at paired behaviors which are in a specific sequential relation to each other. For example, alignment can be analyzed on the speech turn level; that is, one compares the behavior in turn *x* from speaker A and in the following turn *y* from speaker B. Thus in this case adjacent speech turns are taken as the unit of analysis,^5^ where the aligned lexical item or gesture can occur in any position within those turns (e.g., Fusaroli et al., 2017, for lexical alignment in free interaction and Map Task interactions). It is also possible to look at adjacent behavior independent of speech turns, for example, by comparing a gesture that depicts a particular object with the next gesture that is produced (by the other speaker) to depict that same object in a spot‐the‐differences game (Oben & Brône, 2016). Hence, behaviors are “grouped” based on their sequential relation—in this case, adjacency. Note, as mentioned earlier, that this is related to the dimension time, because sequential adjacency usually implicates a relatively short temporal distance between the two pair parts.

It is also possible to completely abstract away from sequential structure, for instance by simply comparing all instances of a kind of behavior category (such as iconic gestures) from both interlocutors (cf. Bergmann & Kopp, 2012; Louwerse et al., 2012) or by restricting analyses to predefined time windows (Oben, 2015). Such approaches lend themselves to large‐scale quantification at the cost of losing sight of fine‐grained sequential dependencies in the data.

Sequence can also be used as a measurement criterion, by taking any set of paired words or paired co‐speech gestures, and investigate or “measure” their sequential relation. For instance in Chui (2014), co‐speech gestures were investigated in terms of their sequential position, by dividing each stretch of talk into three different “phases”—a presentation, collaboration, and acceptance phase (see also Holler & Wilkin, 2011, for a similar approach). Identifying the sequential relation of paired behaviors is mostly done by those who see alignment as an interactive grounding process. Qualitative work in this tradition has shown that immediate repetition of words in the following turn could be used to initiate repair, express surprise, answer a question, or accept a formulation, to name a few (Dingemanse et al., 2014; Norrick, 1987; Rossi, 2020). Quantitative work has confirmed this for the sequential environment of interactive repair: There is a significantly larger likelihood of finding alignment in adjacent turns in *repair* sequences (consisting of a problematic turn followed by a repair‐initiation) compared to other adjacent turns (Fusaroli et al., 2017). Such repair sequences, which are quite frequent, show that some forms of alignment can be the result of explicit coordination. In contrast, from a priming perspective that sees alignment as low‐level and automated, sequential structures of the discourse would be deemed irrelevant.

Analytical approaches are shaped by research traditions and theoretical stances. In fact, we could derive opposing predictions from the two theoretical accounts: Whereas based on priming accounts we would expect equal amounts of alignment across turn pairs irrespective of sequential organization (as long as the temporal distance is the same), based on grounding accounts we could expect higher amounts of alignment in turns that stand in a specific sequential relation to each other (e.g., repair sequences). Besides hypotheses related to repair or adjacency, from a grounding perspective one could also expect to find more alignment *within* a project or course of action rather than across such sequential boundaries, while from a priming perspective one would again hypothesize equal amounts (as long the temporal distance is matched). Different levels of behavior may be differentially susceptible to sequential organization. Here we have an interesting test bed for contrasting or conciliating priming and grounding approaches, with ample opportunities for new research.

### 4.3 Meaning

The meaning dimension captures the observation that paired behaviors which have a clear relation in terms of time, sequence, and/or form might not always overlap in terms of their meaning. Especially in challenging communicative situations, such as a Maze Task, identical words are sometimes used to denote different things (Garrod & Anderson, 1987; Mills & Healey, 2008). With respect to co‐speech gestures, it is evident that they are highly context‐dependent and two similar gestures can mean completely different things in distinct contexts. Hence, this dimension is an important characteristic of lexical and gestural alignment, but in contrast to the other dimensions, generalizes less well to alignment of linguistic behavior at lower levels and bodily behaviors (most of which do not convey semantic meaning).

Seeing meaning as a separate dimension also helps to differentiate lexical and gestural alignment from the notions of *semantic alignment* (e.g., Dideriksen, Fusaroli, Tylén, Dingemanse, & Christiansen, 2019) and *semantic co‐ordination* (e.g., Garrod & Anderson, 1987). Lexical and gestural alignment are generally understood as the repetition of words or gestures independent of the meaning conveyed; in terms of our framework, form is privileged over meaning. A possible empirical approach in line with this notion of alignment is to search for cross‐participant repetition of (lemmatized) words in transcripts (cf. Fusaroli et al., 2017; and the Python package ALIGN by Duran, Paxton, & Fusaroli, 2019) or to measure the form similarity of gestures (e.g., Bergmann & Kopp, 2012). While degree of form overlap is empirically observable, for comparing meanings we must rely on inferences and contextual anchoring. One approach is to manually code for semantic relations between lexical behaviors, for example, by categorizing phrases into “families” of confidence expressions in a joint perceptual task (Fusaroli et al., 2012) or into “mental models” of maze configurations in a maze game (Garrod & Anderson, 1987). More automated measures of semantic relations have also been employed recently, such as the use of word embeddings in a high‐dimensional semantic space (Dideriksen et al., 2019; Duran et al., 2019) or conceptual/semantic recurrence quantification analysis (Angus & Wiles, 2018).

The meaning dimension can also be used as an additional grouping or selection variable in studies on lexical and gestural alignment. For example, in a study by Holler and Wilkin (2011), iconic or metaphoric gestures are only considered to be aligned (in their terms: “mimicked”) when they have some similarity in their form *and* represent the same meaning. Similarly, in Oben and Brône (2016), words or gestures are only considered to be aligned when they refer to the same referent in a spot‐the‐difference game. This is in sharp contrast with, for example, Louwerse et al. (2012), where alignment is operationalized as mere formal similarity in some time window, without reference to meaning (e.g., two gestures are considered to be “matched” when they are both *deictic* gestures, irrespective of the referent that was pointed to).

There are various ways to examine the semantic overlap between instances of behavior, which is often far from trivial. In qualitative studies on lexical or gestural alignment in free conversation (e.g., Kimbara, 2006; Tabensky, 2001; Tannen, 1989), researchers rely on the discourse context to know whether the interlocutors are referring to the same thing or just happen to use the same word or gesture to denote something else. Task‐based approaches have the benefit that the researchers can experimentally control and keep track of the referents that the participants verbally or gesturally refer to. Examples are Brennan and Clark (1996), Clark and Wilkes‐Gibbs (1986), and Holler and Wilkin (2011); in these studies, participants refer to objects on cards, over multiple rounds, which enables the researchers to track the referring expressions to particular objects over longer distances of time. However, there is rarely an exhaustive correspondence between the semantics of words or gestures and the referent they signify. This is because participants can talk about the *same referent*, yet lexically or gesturally single out different semantic properties; for example, when using the word “straight” or a gesture to depict the orientation versus shape of (a part of) an object. And conversely, words or gestures about *different referents* could still be semantically related. For example, in matching tasks with tangram figures, participants might lexically align on basic‐level categories such as heads, arms, etc., which they apply to all stimulus items (Bangerter, Mayor, & Knutsen, 2020).

In the monolingual spoken or written settings most often studied in psycholinguistics, the meaning and form dimensions of alignment can be hard to disentangle. We have highlighted here the potential of multimodal interaction for investigating semantic convergence and divergence. Multilingual interaction (Byun, de Vos, Zeshan, & Levinson, 2019; Costa, Pickering, & Sorace, 2008; Gries & Kootstra, 2017; Schneider, Ramirez‐Aristizabal, Gavilan, & Kello, 2020) offers another promising and understudied environment in which these dimensions can be teased apart to varying degrees.

The meaning dimension draws the clearest line between the priming and grounding approaches. Priming approaches argue that a low‐level, automatic mechanism results in form overlap in behavior, which can lead (or “percolate”) to alignment of semantic representations or vice versa, without semantics as a necessary guiding factor. Grounding approaches, on the other hand, regard instances of behavior as means to negotiate and calibrate mutual understanding, so they expect alignment to occur when there is a semantic or referential link between the instances of behavior.

### 4.4 Form

The form dimension in our framework reflects the fact that some degree of form similarity is the sine qua non of most notions of behavioral alignment in the literature. Lexical and gestural alignment can occur in various ways. For example, with respect to lexical alignment, interlocutors can repeat their partner’s words or phrase literally, or repeat with variation, such as turning a statement into a question or vice versa (Fusaroli et al., 2017; Tannen, 1989). Though some also consider rephrasing or paraphrasing to be forms of “repetition” (e.g., Tabensky, 2001; Tannen, 1989) or “linguistic alignment” (Fusaroli et al., 2012), most studies adopt a more conservative notion of lexical alignment, requiring the repetition of a particular base word or lemma, thus excluding synonyms or paraphrases (cf. Fusaroli et al., 2017; Howes et al., 2010; Oben & Brône, 2016). However, studies vary considerably in the units of comparison; whereas some work with complete speech turns (Fusaroli et al., 2017), others only include content words (Brennan & Clark, 1996), or even a more restricted subset such as high‐frequency words (Nenkova et al., 2008), nouns and verbs (Bangerter et al., 2020), or only nouns (Oben, 2015).^6^ Obviously the degree of detected alignment can differ dramatically as a function of which terms are included in the comparison.

Similar to lexical alignment, “gestural rephrasing” has also been considered as a form of “repetition” in the gestural modality (e.g., Tabensky, 2001). However, most studies on gestural alignment require at least some degree of form resemblance, though studies vary with respect to how this is measured. Studies focusing on gestural form similarity generally analyze *iconic* co‐speech gestures, which are spontaneous, idiosyncratic gestures where plenty of variations in form are possible. This is in contrast to *deictic* gestures (i.e., pointing gestures), *emblems* (such as the thumbs‐up gesture), and *interactive* gestures (such as *beats* or *palm‐up open‐hand* gestures), which have more conventionalized forms. Most studies on gestural alignment are based on manual coding, where form overlap has been operationalized in terms of *mode of representation* (or representation technique, e.g., the hands can draw the outline of an object, enact a certain action, etc.; Streeck, 2008), specific form features or a combination of those. Recent advances in the field point to the promise of automated measures for quantifying the kinematic resemblance of gestures in terms of their velocity, size, distance, etc. (Pouw & Dixon, 2020).

**Fig. 3:**
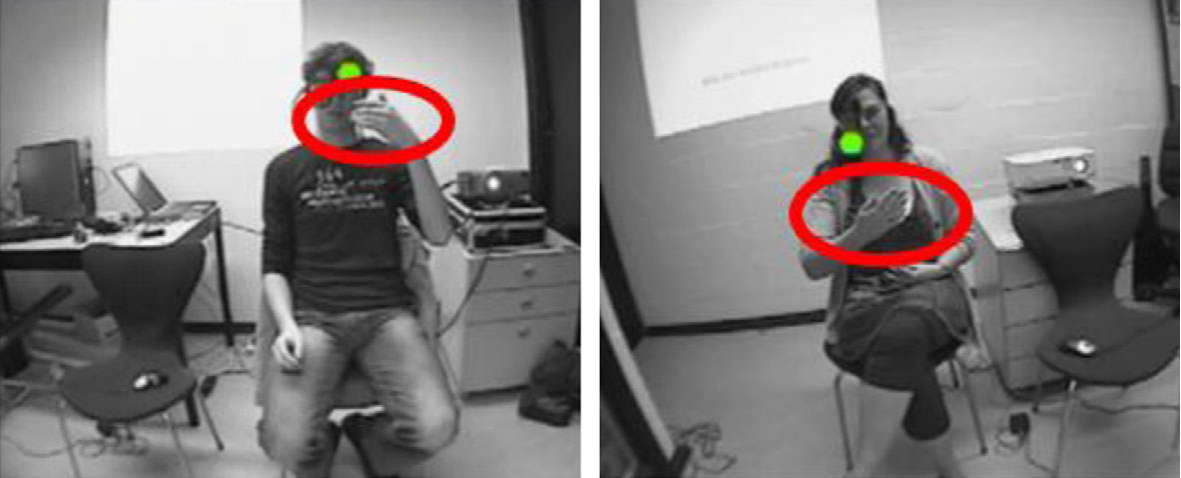
Gestures with overlap in “modeling” as the mode of representation. Reproduced with permission from Oben and Brône (2016).

Several studies on gestural alignment use mode of representation as a grouping variable. Oben and Brône (2016) used overlap in mode of representation as their primary criterion for considering gestures to be aligned (thus ignoring such features as motion or position), while Holler and Wilkin (2011) used it along with the requirement to have the same overall shape/form (where some variability in handshape or position was accepted, but not in handedness). As an example of how mode of representation is used as a criterion, see Fig. 3.

Here, both participants gesturally depict the target object DOOR, where the hand is a “model” for the object. The gestures differ in terms of handedness, finger orientation, and the tension in the handshape. However, Oben and Brône (2016) “still consider it to be an instance of gestural alignment because the representation technique is identical (i.e. modelling)” (2016, p. 37).

**Fig. 4:**
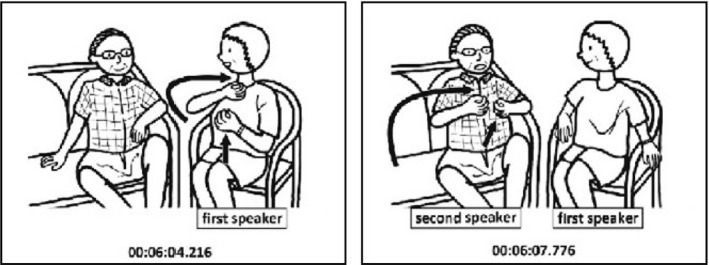
Gestures with overlap in handedness, handshape, position, and motion. Reproduced with permission from Chui (2014).

The other approach is to compare gestures on a number of form features. For example, Bergmann and Kopp (2012) investigated gestural alignment separately for mode of representation and other form features (handedness, handshape, palm‐ and finger orientation, and wrist movement type). Chui (2014) coded whether gestures overlapped in terms of handedness, handshape, position, motion, and orientation. Of the 12 gesture pairs in the analyses that were identified as “mimicked”, 11 pairs showed overlap in four or five form features, and one pair in three features. See, for example, the following gesture pair (Fig. 4).

Here, both speakers gesturally depict a musical instrument: Both use two hands (overlap in handedness), with the fingers curled into fists (overlap in handshape), facing each other in front of the chest (overlap in position), moving one hand to enact the idea of moving a bow (overlap in motion). However, as Chui notes, there is some deviance in the orientation of the lower hand (as the second speaker rests his arm on the sofa). She concludes that “in considering the five features together, the deviance in the hand/finger orientation, but the high consistency in the other four features did not affect the conclusion of the analysis that the two gestures were highly similar gestures for the same referent” (p. 73).

Both priming and grounding perspectives use form as their main criterion for considering behavior to be aligned. However, there are important differences in the role form overlap plays from a theoretical point of view. Whereas priming is considered to naturally result in form overlap (due to activation of motor plans or linguistic representations), grounding perspectives consider more explicit coordination to (also) play a role. Furthermore, as discussed in Section 2, under grounding accounts there is no necessary relationship between alignment of behavior in terms of form, on the one hand, and mutual understanding, on the other—as understanding can also be achieved through repetitions with variations in form, or even through the production of completely different words or gestures altogether rather than aligning. Consequently, those working from a priming perspective might apply stricter form criteria for selecting paired behaviors than those working from the grounding perspective.

### 4.5 Modality

Behaviors can differ in the mode in which they are produced and perceived. For instance, they may be auditory‐vocal behaviors, like spoken words, or visual‐gestural behaviors, like signs and gestures (Meier et al. 2002). A prevalent assumption in the work on alignment is that for any pair of behavior which is considered aligned, the behavior is produced within the *same* modality. That is, the relation between the two pair parts of behavior is considered to be a unimodal one. However, from a theoretical point of view, the two parts of aligned behavior can also be in a *cross‐modal* relation to each other, as long as they are aligned on one or more of the other five dimensions. This is less intuitive, presumably because of the (implicit) assumption that behavior should be similar in form to at least some degree, which is difficult when produced in different modalities. However, we argue that two instances of behavior, which are in a certain sequential, temporal, and/or meaning relation to each other, can still be considered aligned.

Though not considered in the original model of Pickering and Garrod (2004), there is evidence that iconic gestures can prime semantically related words (e.g., Yap, So, Yap, Tan, & Teoh, 2011), which would be a form of cross‐modal alignment coming about through priming. From a grounding perspective, cross‐modal alignment could be employed for communicative purposes, since lexical and gestural representations have been shown to be linked at the conceptual level (Mol et al., 2012). Both approaches thus build on the assumption that the matching of public behavior in interaction ultimately must be related to some sort of convergence in private conceptualizations (at least for communicative speech and gesture). However, this implies that an instance of cross‐modal alignment can only be identified on the assumption that we can identify a common conceptual thread to what people are communicating about—which can be challenging, especially in free conversation. To our knowledge, there is only one study, which has investigated lexical and gestural alignment with such a cross‐modal approach. Tabensky (2001) investigated free conversations and reports interesting cases of what could be denoted as cross‐modal alignment: Certain semantic information, which was initially conveyed verbally by one person, can be repeated by means of gestures by the other person, and vice versa. Take the following example (English translation, simplified transcription) from a conversation between two speakers (D and N) about buying a house:

**Table:** 

(3)		D	a flat is‐ unless it measures [a hundred and eighty square meters]
		N	yeah like [a duplex or something]

D aims to convey the size of a big apartment; he produces the lexical phrase “a hundred and eighty square meters” and simultaneously makes a gesture by opening and separating his hands sideward, while also raising his chin. Tabensky argues that this gesture conveys additional semantic information, which is not expressed in speech; that is, the gesture conveys both width and height. His conversation partner N takes up the information from the two modalities, and subsequently repeats both idea units in a new lexical phrase: “a duplex” (i.e., a spacious apartment on two levels). In Tabensky’s words, she was “verbally re‐encoding the sum of information she has just been offered by way of two simultaneous modes of communication” (2001, p. 221).

Work on alignment from a cross‐modal perspective is scarce, and cases of cross‐speaker gesture–speech alignment have been overlooked in studies restricting their analyses to alignment in either gesture or speech. However, this is not to say that alignment has not been approached from a multimodal perspective at all. It has been explored in a different way, as researchers have investigated how alignment within one modality relates to alignment within another modality. Specifically, they aim to find out whether alignment of various types of behavior or linguistic levels are driven by the same underlying mechanisms and serve similar functions, or are in fact independent phenomena at different levels of processing. For example, the interactive alignment model “assumes interrelations between all levels” (p. 183) and proposes that “interlocutors will tend to align expressions at many different levels at the same time” (Pickering & Garrod, 2004, p. 175). Though their model is centered on speech, it could be extended to include co‐speech gestures. There are two empirical studies which have investigated such interrelations for speech and gesture—Louwerse et al. (2012) and Oben and Brône (2016)—which we will discuss in turn.

In Louwerse et al. (2012), many kinds of behavior in multiple modalities (linguistic expressions, facial expressions, manual gestures, and noncommunicative postures) were found to be aligned in form and time. The authors furthermore argue that “the mechanisms underlying this widespread synchronization seem to have a unitary character, given the simultaneous modulation of the synchrony in our results” (p. 1423). In Oben and Brône’s (2016) study, participants engaged in a spot‐the‐difference game, in which they had to refer to various objects in animated videos. Lexical and gestural alignment were operationalized as adjacent references to the objects produced by the two speakers, which overlap in root form (for words) or mode of representation (for gestures). They found no correlation between the two kinds of alignment; “target objects that are often lexically aligned are not systematically gesturally aligned as well” (p. 41). Furthermore, they found that lexical and gestural alignment can be explained by different factors: Lexical alignment is predicted by the number of times one’s conversational partner has used a word, whereas for gestural alignment temporal overlap in referring to an object (i.e., whether or not a gesture was produced simultaneously or with a lag) is the most important factor. Thus, in contrast to Louwerse et al. (2012), Oben and Brône (2016) conclude that lexical and gestural alignment seem to be governed by different rules.

With the exceptions of these two studies, most investigations into lexical alignment have adopted a strictly unimodal perspective, where nonverbal aspects of interactions were not taken into account (note that commonly the task setting was such that participants could not see each other; e.g., Brennan & Clark, 1996; Garrod & Anderson, 1987). On the other hand, studies of gestural alignment generally do elaborate on the relation between gestures and the accompanying speech, yet lack a systematic investigation of lexical alignment. For example, Holler and Wilkin (2011) descriptively distinguish between various ways in which gestural alignment relates to speech. They note that gestural alignment is often accompanied by lexical alignment (e.g., consistently referring to a figure as “the ice skater,” along with a physical reenactment), resulting in so‐called conceptual pacts. Yet such coinciding lexical alignment does not always occur, as gestural alignment can also be sufficient on its own to effectively refer to an object or to express acceptance of that reference. Holler and Wilkin report cases of strong gestural convergence which “carry most of the communicational burden,” thereby eliminating the need for lexical alignment, and allowing for less precision and more cross‐speaker variation in verbal referring expressions. For example, members of a dyad could interchangeably refer to a figure as either having “arms” or “things” sticking out, yet be consistent in the use of the accompanying gesture (two arms representing the position of the figure’s arms). These observations are in line with the findings of Tabensky (2001) and Chui (2014), who found that interlocutors can repeat a certain gesture while producing a verbal description which diverges from their speech partner’s, thus putting the gesture into a new relationship to speech.

In terms of theory‐based hypotheses, priming accounts expect alignment to be linked across (linguistic/conceptual) levels, which might generalize to links across multiple modalities. Hence, similar to how lexical and semantic alignment seem to “boost” syntactic alignment (Branigan et al., 2000; Cleland & Pickering, 2003; Mahowald et al., 2016), lexical and gestural alignment could also be predicted to go hand in hand. However, according to grounding accounts, the relation between modalities might be flexibly adapted to the specific communicative needs at hand—for example, by aligning in the manual modality, while purposefully misaligning lexically, or vice versa. So, from a grounding perspective, cross‐modal alignment may, but need not, occur, and the division of labor between gesture and speech may be manipulated for communicative or coordinative effect.

### 4.6 Review summary

By unpacking notions of alignment into five distinct dimensions, each of them independently motivated and grounded in empirical work, we have characterized the space of possibilities in which operationalizations of alignment can be situated and compared. We distinguished two prominent theoretical perspectives (*priming* and *grounding*) and showed how their assumptions regarding the underlying mechanisms of alignment pattern together with methodological choices and empirical foci. A summary of the two perspectives is presented in Table 2.

**Table 2:** Schematic summary of relations between empirical and theoretical approaches

	Priming	Grounding
Underlying mechanism	Automatic Non‐intentional Low‐level	Controlled Intentional Higher level
Data collection	Controlled experiments Task‐based interactions	Naturalistic interactions Task‐based interactions
Modes of analysis	Quantitative	Qualitative
Dimensions prioritized	Time Form	Sequence Form Meaning

Broadly speaking, studies that are premised on the notion that communication is (at least partly) driven by automatic, lower‐level processes (the priming approach) tend to consist of quantitative analyses to compare instances of behavior irrespective of their sequential relation, prioritize form resemblance (rather than meaning overlap), and restrict analyses to one modality. In contrast, the line of work in which communication is regarded as an interactive, collaborative undertaking (the grounding approach) is more likely to involve qualitative analyses, with a focus on semantic information conveyed by the potentially aligned behavior, paired with a consideration of the (multimodal) discourse context and its sequential structure.

It bears repeating that priming and grounding merely represent two points of attraction in a larger space of possibilities. We tabulate them here to bring to light what is perhaps a growing tendency in current strands of work to align with one or the other and favor distinct sets of mechanisms, methods, and analyses.^7^ However, as our framework shows, it is possible (and indeed perhaps desirable) to carry out fundamental work on behavioral alignment while taking inspiration from across these perspectives. The five omnirelevant dimensions of alignment that make up our integrative framework are designed to facilitate such research.

As our summary shows, the five dimensions differ in terms of their relative importance. The form dimension seems to be most prominent in the literature, understandably since this is the most directly observable (though operationalizations vary). The dimensions of time and meaning are also deemed important; priming accounts predict that priming effects decrease over time, and work from a grounding perspective tends to consider behaviors to be aligned only when they also involve shared meaning. However, our review of the literature shows that there is as yet limited theoretical and empirical work with respect to the dimensions of sequence and modality—yielding promising avenues for future research.

## 5 Discussion

There is an ever‐expanding line of research on alignment in interaction, with a broad range of theoretical and empirical approaches. We demonstrated that seemingly related studies have very different approaches to the phenomenon, which are hard to reconcile because they refer to qualitatively different types of alignment. In an effort to enable cumulative progress and principled comparison, we unpacked the complex notion of alignment into five constituent dimensions. We distinguished between *priming* and *grounding* as the two most prominent theoretical perspectives, and showed that priming approaches prioritize the dimensions form and time, while grounding approaches mostly focus on sequence, form, and meaning. In this section, we identify a number of open questions in the field and make suggestions for how the framework can benefit future work.

One opportunity for further research is the relation between forms of alignment at various types and levels of linguistic and communicative behavior. More work is needed to ascertain whether the current postulated underlying mechanisms (priming vs. grounding) generalize to alignment of any behavior, or perhaps only apply to a specific subset. For example, repeating another’s words to resolve a misunderstanding may seem to point in the direction of grounding, whereas alignment in terms of posture might be better explained through priming. Other kinds of behavioral alignment might fall somewhere in between, with strategic as well as more automatic components being at play simultaneously (cf. Kopp & Bergmann, 2013).

Second, more work is needed on the causal relations between alignment at various channels or (linguistic) levels of behavior. From a priming perspective, it has been argued that alignment at one level can “percolate” to other levels (Pickering & Garrod, 2004). There is certainly strong evidence for this with respect to syntactic, lexical, and semantic alignment (see Mahowald et al., 2016, for an overview), though we are not aware of published evidence for the “link‐between‐levels” claim for lower linguistic levels (e.g., phonetics), or across modalities (e.g., lexical choice and co‐speech gestures; Oben & Brône, 2016). From the grounding perspective, one might argue that different kinds of behavioral alignment yield different communicative *affordances* (depending on the task at hand), which could have implications for the order in which they occur. For example, when referring to novel objects or concepts, the use and alignment of iconic co‐speech gestures can (by virtue of their form‐meaning resemblance) constitute a gateway into shared conceptualizations, which might precede any alignment in terms of lexical choice. The qualitative observations from Holler and Wilkin (2011) seem to line up with this reasoning and provide inspiration for follow‐up studies.

Some of the opportunities for new research we have identified here result from the challenges involved in comparing findings on various types and levels of (linguistic) behavior. As shown, there is a large space of empirical possibilities for studying alignment, and design choices in this space are often guided by research traditions and theoretical presuppositions. To make such choices more visible, and to increase the commensurability of work across theoretical perspectives, we recommend that studies clearly explain how alignment has been operationalized and which dimensions have been privileged. The theory‐agnostic framework proposed here can be a useful resource: Adopting a common terminology for the building blocks of alignment will greatly enhance comparability and theory building in the field.

Our overview of the alignment possibility space has also shown a dearth of theoretical and empirical work with respect to the dimensions of sequence and modality. Regarding sequence, many quantitative analyses tend to ignore the inherent sequential structure of (task‐based) interactions altogether. However, this could be an interesting test bed for differentiating between diverging theories. From a grounding perspective, there are good reasons to believe that alignment rates will be higher within certain sequences. In contrast, presuming that an automatic priming mechanism underlies alignment, we could hypothesize that only temporal proximity affects alignment, irrespective of sequential relation.

When it comes to the modality dimension, various theories leave open the possibility of cross‐modal alignment, although empirical evidence is still lacking. Cross‐modal alignment is presumably not considered to be alignment (nor “repetition,” “mimicry,” or “behavior matching”), because there is a lack of form resemblance, which is a key characteristic in both grounding and priming accounts. However, when listeners align to the speaker’s verbal narration in a nonverbal manner, such as wincing or showing a concerned facial expression when someone tells a close‐call story (Bavelas et al., 2000), this could be considered a form of meaning alignment. Yet cross‐speaker speech–gesture relationships remain understudied (Tabensky, 2001), which is remarkable, given that speech and gesture are semantically co‐expressive (McNeill, 1992), and tightly linked in both production and comprehension (Cassell, McNeill, & McCullough, 1998; de Ruiter, Bangerter, & Dings, 2012; Kita & Özyürek, 2003; Kopp & Bergmann, 2013; Mol et al., 2012; for a review, see Özyürek, 2018). Thus, little is known about whether, and if so how, lexical and gestural alignment are interrelated, making it a promising avenue for further research.

In closing, we outline three specific recommendations for work on cross‐participant alignment of communicative behavior:

1. *Theorize alignment phenomena using common conceptual foundations*. Use the dimensions of time, sequence, meaning, form, and modality to delineate alignment, and to formulate theories and testable predictions about its cognitive mechanisms and communicative functions.
2. *Describe operationalizations to enable targeted comparisons*. Explicitly describe *which* instances or streams of behavior are compared and *how* those are compared. That is, describe how behavioral similarities are measured and how observations are selected, grouped, manipulated, or measured in terms of the five dimensions of time, sequence, meaning, form, and modality.
3. *Combine methods to build a more comprehensive view of alignment*. Combine observational and experimental methods, and qualitative and quantitative approaches, to further unravel the multidimensional nature of alignment—especially in terms of sequence and modality, which remain largely unexplored.

Following these recommendations will contribute to increased interdisciplinary coherence, will enhance the reproducibility and generalizability of results, and will enable more principled comparisons across the fields that study the alignment of communicative behavior.

## 6 Conclusion

A paper with the goal of charting different takes on alignment and related phenomena in human interaction cannot escape the ironic observation that there appears to be, on the surface, a relative lack of alignment on basic terminology in related fields that would benefit from working together. However, as argued, even different lexical labels may mask deeper underlying similarities. Here we have sought to bring out the most important of these in terms of five constituent dimensions relevant to any notion of cross‐participant alignment in interaction: time, sequence, meaning, form, and modality.

By decomposing the multidimensional nature of alignment in this way, we have brought into view a wealth of theoretical interpretations and empirical operationalizations of alignment. We hold that no account of alignment in interaction can be complete without explicating the phenomenon in terms of these five dimensions, which crosscut levels of analysis and assumed mechanisms. In time, the rise of explicit operationalizations of alignment and kindred notions in terms of these basic dimensions will result in greater commensurability and comparability of empirical and theoretical work. We hope the framework will be of use as a conceptual tool to disclose hidden assumptions, refine theoretical accounts, and enable cumulative progress in the study of alignment in interaction.

## Notes

^1^ Stills reproduced under fair use from the video “Mila Kunis & Christian Slater are addicted to dating shows” by The Late Late Show with James Corden, 2018 (https://www.youtube.com/watch?v=B2Pcc_CSaK4, 2:40–2:41).

^2^ However, note the difference in terms of handedness: M produces a two‐handed gesture, while C only uses his left hand to illustrate the “eating.” Some might therefore argue that these gestures are not aligned (or not “mimicked” or “matched”). Later in the paper we will further discuss such criteria regarding overlap in form.

^3^ Though such accounts have recently been challenged, for example, by arguing that this kind of automatic mimicry is a flexible and socially guided process (Chartrand & van Baaren, 2009; Lakin & Chartrand, 2003), which operates under top‐down control of the mentalizing system—a system recruited to infer other people’s mental states or to make social judgments (Wang & Hamilton, 2012).

^4^ Though the term "priming" generally refers to a short‐term, automatic effect, a case has also been made for the existence of so‐called “long‐term” priming (cf. Pickering & Garrod, 2004), which has been shown to even persist over the course of a week (Kaschak et al., 2011). See also Reitter et al.’s model (Reitter et al., 2011), which differentiates between short‐term priming and long‐lasting adaption.

^5^ Note that we use the term “adjacency” here in the simple sense of adjacent or neighboring; not to be confused with the Conversation Analytic term *adjacency pairs*, as referred to earlier.

^6^ The exact operationalization of such constructs is not straightforward either. For example, there has been ample debate about what constitutes a speech turn, and how they can be recognized in conversations (Selting, 2000). Referring expressions or noun phrases can also be problematic units of analyses in natural interaction, due to the frequent occurrence of ellipsis and grammatically incomplete utterances.

^7^ Indeed, an anonymous reviewer brings up the possibility that the distinction has been “amplified by dueling labs.”
